# Ca^2+^ Channels Mediate Bidirectional Signaling between Sarcolemma and Sarcoplasmic Reticulum in Muscle Cells

**DOI:** 10.3390/cells9010055

**Published:** 2019-12-24

**Authors:** Guillermo Avila, Juan A. de la Rosa, Adrián Monsalvo-Villegas, María G. Montiel-Jaen

**Affiliations:** Department of Biochemistry, Cinvestav-IPN. AP 14-740, México City, DF 07000, Mexico

**Keywords:** intracellular Ca^2+^, Ca^2+^ channel, contractility, Ca^2+^-induced Ca^2+^ release (CICR), excitation-contraction coupling, ryanodine receptor (RyR), dihydropyridine receptor (DHPR)

## Abstract

The skeletal muscle and myocardial cells present highly specialized structures; for example, the close interaction between the sarcoplasmic reticulum (SR) and mitochondria—responsible for excitation-metabolism coupling—and the junction that connects the SR with T-tubules, critical for excitation-contraction (EC) coupling. The mechanisms that underlie EC coupling in these two cell types, however, are fundamentally distinct. They involve the differential expression of Ca^2+^ channel subtypes: Ca_V_1.1 and RyR1 (skeletal), vs. Ca_V_1.2 and RyR2 (cardiac). The Ca_V_ channels transform action potentials into elevations of cytosolic Ca^2+^, by activating RyRs and thus promoting SR Ca^2+^ release. The high levels of Ca^2+^, in turn, stimulate not only the contractile machinery but also the generation of mitochondrial reactive oxygen species (ROS). This forward signaling is reciprocally regulated by the following feedback mechanisms: Ca^2+^-dependent inactivation (of Ca^2+^ channels), the recruitment of Na^+^/Ca^2+^ exchanger activity, and oxidative changes in ion channels and transporters. Here, we summarize both well-established concepts and recent advances that have contributed to a better understanding of the molecular mechanisms involved in this bidirectional signaling.

## 1. Introduction

In striated muscle, chemical energy is converted into physical work. The skeletal muscle is responsible for breathing, retaining the posture, and locomotion, whereas the cardiac muscle is essential for blood pumping. The primary basis for energy conversion relies on adenosine triphosphate (ATP) hydrolysis and a delicate interplay between components of the contractile machinery, whose dependence on cytosolic Ca^2+^ is also of critical relevance (particularly for controlling the shortening-relengthening cycle [1]). Thus, Ca^2+^ is the commander for this chemical–physical transformation, and thus its concentration is not arbitrarily set. Instead, the homeostasis of Ca^2+^ is governed by Ca^2+^ handling proteins, such as channels, transporters, and ATPases. For example, the source of Ca^2+^ for activating the contractile machinery is the sarcoplasmic reticulum (SR), which is loaded thanks to the activity of the sarcoplasmic reticulum Ca^2+^-ATPase (SERCA [2]). 

The SR can be divided into three different regions: junctional, which projects towards the sarcolemmal transverse (T) tubules and contains ryanodine receptors (RyRs); network, which expands throughout the sarcomere and surrounds the myofibrils; corbular, an extension of the network SR but that is confined to regions away from the sarcolemma and also contains RyRs. The T-tubules contain a subgroup of voltage-gated Ca^2+^ channels (VGCCs), termed dihydropyridine receptors (DHPRs), which connect the firing of action potentials (APs) with the activation of RyRs, and thereby promote SR Ca^2+^ release and muscle contraction. More precisely, the SR Ca^2+^ release is finely tuned by Ca^2+^ release units: that is, interacting clusters of DHPRs and RyRs (also known as couplons [3]). These regions are enriched with other ion channels and second messengers, and, thus, they represent key signaling hubs for the regulation of muscle function. The T-tubules ensure the propagation of APs into the central regions of muscle fibers, allowing Ca^2+^ release to be synchronous throughout the entire cytoplasm. One T-tubule is surrounded by one or even two SR membranes, forming specialized regions termed dyads and triads [3,4]. 

Here, we discuss the critical role of DHPRs in mediating the forward and backward signaling between the sarcolemma and the SR, along with the contribution of other molecules involved. In the last section, the SR-mitochondria interaction is also briefly reviewed. The number of diseases related to alterations in the couplon is vast, and thus we only revise few and emblematic disorders. Due to space limitations, many original studies were not cited. Thus, we encourage readers to consult other recent and influential reviews. An abbreviations list was included to improve readability (see Abbreviations). 

## 2. Voltage-Gated Ca^2+^ Channels (VGCCs)

Voltage-gated Ca^2+^ channels (VGCCs) are oligomer complexes that consist of the following subunits: α_1_, β, α_2_, δ, and γ [5,6,7]. In mammals, ten genes encode for distinct α_1_ subunits. They contain both the pore region and voltage sensors, whereas the other (accessory) subunits modulate the gating and surface expression of α_1_. Skeletal muscle fibers and cardiac myocytes primordially express Ca_V_1.1 (α_1S_) and Ca_V_1.2 (α_1C_), respectively [8,9]. The α_1_ subunit consists of four homologous transmembrane repeats (domains I-IV), which contain six transmembrane segments (S1–S6). S4 includes positively charged residues and thus acts as the voltage sensor. The channel pore is formed by segments S5 and S6, as well as their linking loop. The linkers between each domain and the N- and C-terminal fragments are cytosolic and interact with other regulatory proteins [5,6,7]. The Ca_V_1.1 and Ca_V_1.2 channels exhibit a high affinity for dihydropyridines (DHPs); thereby, they are also known as DHP receptors (DHPRs). β is probably the most important accessory subunit, because it regulates not only the gating and surface density of α_1_, but also the skeletal muscle EC coupling [10]. 

## 3. Ryanodine Receptors (RYRs)

The three known mammalian isoforms of RyRs (RyR1, RyR2, and RyR3) share a high percentage of identity, particularly in their C-terminal region, which also contains transmembrane sections that shape the selectivity filter of the channel. They are tetramers, and their cytoplasmic portion (~90%) is large enough (approximately two kD) to be seen under electronic microscopy (electrodense images termed feet). This region represents a convergence point for multiple regulatory signals, which range from metabolites and ions, to protein kinases and accessory subunits [11]. 

The selectivity filter of RyRs is composed of a peptide sequence of three amino acids (GYG), which is highly conserved not only between other intracellular Ca^2+^ release channels like inositol trisphosphate receptors (GVG) but also voltage-gated K channels (GYG). Unsurprisingly, this domain is located in the C-terminal fragment, which contains the “pore loop,” transmembrane segments, and the luminal domain [12,13,14]. The latter senses levels of luminal Ca^2+^ [15] and interacts with other proteins of the SR (i.e., triadin, junctin, and calsequestrin), which modify the gating of the channel [16,17] and, thereby, also influence EC coupling [18,19]. Although the RyRs have not yet been crystallized, their architecture has been revealed in great detail, thanks to the astonishing 3D images obtained with cryo-electron microscopy [20]. For example, the 3D structure of rabbit RyR1 has been resolved at near-atomic resolutions of 3.8 Å [21,22,23]. 

The open probability (P_o_) of RyRs exhibits a bell-shaped dependence of the intracellular concentration of calcium ([Ca^2+^]_i_). It begins to increase at nearly 1 µM, reaches the maximum at 100 µM, and then gradually decreases with concentrations in the millimolar range. This biphasic behavior is likely explained by the existence of two possible Ca^2+^-binding sites, of high and low affinity: the former could generate activation (i.e., Ca^2+^-induced Ca^2+^ release (CICR)) and the latter inhibition. Indeed, the inhibition of RyRs by high [Ca^2+^]_i_ contributes to ending the positive feedback inherent to CICR [24,25,26].

## 4. Skeletal Muscle EC Coupling

### 4.1. The Skeletal-Type EC Coupling Depends on a Ca_V_1.1-RyR1 Physical Interaction

**Figure 1:**
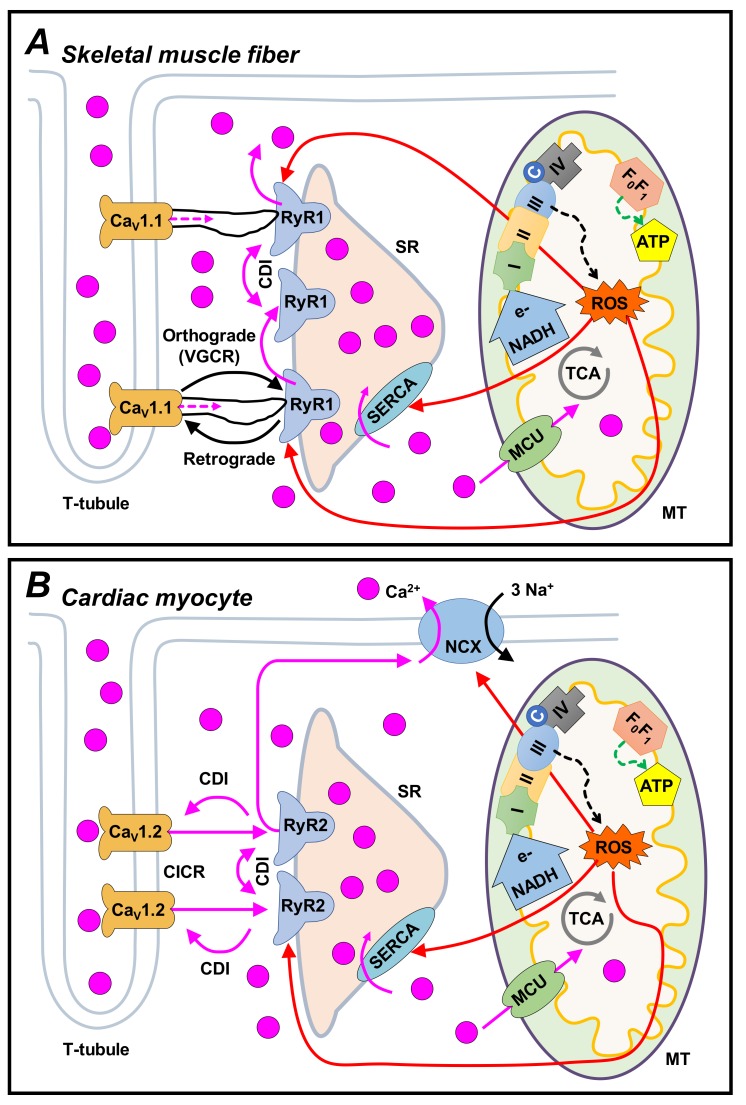
The voltage-gated Ca^2+^ channels (VGCCs) govern feedback mechanisms of the T-tubule-SR junction and, thereby, also influence the SR-mitochondria communication. The figure illustrates the principal bidirectional signaling pathways that operate in skeletal (**A**) and cardiac (**B**) muscle. In both cases, an action potential (AP) activates Ca^2+^ channels of the sarcolemma (*Ca_V_1.1* and *Ca_V_1.2*), which promotes SR Ca^2+^ release via RyRs (*RyR1* and *RyR2*), in a process known as EC coupling (*orthograde* signaling). The underlying mechanisms, however, are distinct. In the latter, the Ca_V_1.2 to RyR2 communication consists in Ca^2+^-induced Ca^2+^ release (*CICR*), whereas in the former, the Ca_V_1.1 channels directly activate RyR1s, thanks to a physical link (Ca^2+^ is not required, and, thereby, this phenomenon is also known as voltage-gated SR Ca^2+^ release, *VGCR*). The rise in [Ca^2+^]_i_ activates the SERCA pump and the NCX, returning [Ca^2+^]_i_ to baseline levels—with the aid of Ca^2+^-dependent inactivation of both RyRs and Ca_V_1.2 (*CDI*). A small portion of Ca^2+^ ions permeates into the mitochondrion (MT), e.g., via the mitochondrial Ca^2+^ uniporter (MCU). Then, a symbiotic relationship between the SR and mitochondria occurs, because the calcium ions stimulate the synthesis of ATP, which is required for not only SERCA activity but also the cross-bridge cycle of contraction. In parallel, the tricarboxylic acid (TCA) cycle generates reducing equivalents (e.g., NADH) which are transferred to the electron transport chain (complexes I–IV), whose activity produces superoxide ion (O^−^, dashed black line) which, in turn, is converted into H_2_O_2_. The latter is a substrate for the Fenton reaction (forming hydroxyl radical, OH^-^). These reactive oxygen species (ROS) can react with (and modulate) the EC-coupling related proteins (red lines), creating a delicate balance that contributes to optimal muscle performance. Nevertheless, an excessive rate of ROS generation can lead to severe oxidative damage, EC uncoupling, and cell death.

In skeletal muscle, the entry of extracellular Ca^2+^ is not required for EC coupling (Figure 1A, dashed pink line). For example, the skeletal fiber contracts for several minutes in the absence of extracellular Ca^2+^ [27]. Besides, EC coupling prevails following the pharmacological blockade of Ca^2+^ influx [28,29], and is unaltered by a pore mutant of Ca_V_1.1 that disrupts Ca^2+^ conductance [30,31]. Additionally, both muscle contractions and SR Ca^2+^ release persist at membrane potentials where the driving force for the entry of Ca^2+^ is practically nonexistent [32,33]. 

The well-known “plunger” hypothesis states that voltage sensors of the T-tubules are physically connected to a “plug” in the SR. Conceivably, the voltage sensors move in response to action potentials, and this promotes the release of Ca^2+^ from the SR which, in turn, elicits contraction [34]. Substantial evidence indicates that Ca_V_1.1 and RyR1 are the voltage sensor [35,36] and the SR release channel [37,38], respectively. Thus, in skeletal muscle, EC coupling depends on a process known as voltage-gated Ca^2+^ release (VGCR, Figure 1A). Indeed, Ca_V_1.1 is almost exclusively expressed in skeletal muscle fibers, and generally is not co-expressed with other VGCCs (Ca_V_3.2 has also been detected, but only during ontogeny [39,40]).

In the sarcolemma, the Ca_V_1.1 channels are grouped into tetrads, and each channel is thought to interact with one of the RyR1 monomers physically (i.e., Ca_V_1.1-RyR1 interaction). This arrangement, however, is only present in alternate ryanodine receptors, which results in a checkerboard pattern [41]. Accordingly, the RyR1s can be divided into those physically, and those non-physically bound to Ca_V_1.1. Recently, they were named as V channels (linked to the voltage sensor, Ca_V_1.1) and C channels (assumed Ca^2+^ activation) [42]. 

Within a couplon, the activation of RyRs is controlled locally. That is, the release of Ca^2+^ at each particular cluster of RyRs can be induced by nearby DHPRs, and does not necessarily activate contiguous clusters—due to space limitations and the inactivation of C channels (which interrupts the propagation of Ca^2+^-mediated activity [43,44]). The corresponding elementary Ca^2+^ release events (sparks [45]) occur stochastically and can be synchronized during membrane depolarization to produce a uniform Ca^2+^ release (or global Ca^2+^ transient [46]). 

In addition to RyR1, the skeletal muscle expresses RyR3 [47] (conversely, cardiac myocytes primarily express RyR2 [48]). Only RyR1 can interact with Ca_V_1.1 physically, and thus this particular RyR isoform is critical for skeletal-type EC coupling. Accordingly, the role of RyR3 in skeletal muscle is considered secondary and may only consist of amplifying the release of Ca^2+^ from the SR. Interestingly, however, a recent study showed that this isoform is important for the proper relaxation of extraocular muscle fibers, and, thereby, vision is severely impaired in RyR3 knockout mice [49]. 

Many studies have been devoted to discovering the Ca_V_1.1 segments and accessory proteins that interact with RyR1. Dysgenic (Ca_V_1.1 knockout) myotubes were very helpful in this respect [50]. For example, when artificially expressed in these cells, only L-type Ca^2+^ channels are correctly targeted to the triads (as opposed to Ca_V_2.1 and Ca_V_2.2 channels). Nevertheless, the Ca_V_1.1 channels are unique in being able to form tetrads, suggesting that although Ca_V_1 channels share a common motif which is responsible for proper targeting to the junctions, only Ca_V_1.1 binds to RyR1 [51,52].

Interestingly, the use of Ca_V_1.2-based chimeras led to the discovery that the II–III linker of Ca_V_1.1 (residues 711–765, mainly) is critical for attaining skeletal-type EC coupling and thus also for binding to RyR1 [52,53,54]). Accordingly, synthetic peptides that mimic this linker can interact with RyRs that have been reconstituted into lipid bilayers [55,56,57]. 

With regard to accessory subunits, studies performed on myotubes derived from β_1a_ knockout mice have proposed that this subunit is also involved in EC coupling (for review, see Coronado et al., [58]). Indeed, the absence of β_1a_ results in not only the significant inhibition of VGCR, but also a barely detectable presence of the Ca_V_1.1 channels in the plasma membrane [59]. Given that this downregulation of VGCR can be restored by the expression of exogenous β_1a_, but not β_2a_ [60], the domain of β_1a_ responsible for EC coupling was narrowed using chimeras of these two proteins. The corresponding results suggest that a hydrophobic C-terminal heptad repeat is involved in recapitulating skeletal-type EC coupling [61]. Accordingly, small peptides corresponding to short segments of the β_1a_ C-terminal tail can attach to the RyR1, and this promotes the activity of the latter [62,63]. Furthermore, the acute microinjection of β_1a_ into muscle fibers upregulates EC coupling, whereas a β_1a_ mutant protein with deleted C-terminal domain does not mimic this effect [64]. Therefore, it seems like the β_1a_ subunits dynamically bind and unbind from the EC coupling complex, and the presence of exogenous subunits shifts the corresponding equilibrium towards more complexes with attached subunits. Moreover, Roger Bannister’s group demonstrated that the EC coupling of skeletal muscle fibers is downregulated by the overexpression a protein that binds β subunits (REM), and this effect can be explained by the possible removal of β_1a_ from the Ca_V_1.1-RyR1 complex [65]. 

### 4.2. Role of Other Triad Proteins

STACs are a small family of three members (STAC1, STAC2, and STAC3) of adaptor proteins that facilitate the interaction between protein-binding partners [66]. STAC3 is mainly expressed in skeletal muscle, whereas STAC1 and STAC2 are ubiquitously expressed [67]. Recently, it was found that STAC3 is required for a functional membrane expression of Ca_V_1.1 in both heterologous expression systems and T-tubules [68,69]. Moreover, STAC3 knock-out mice and STAC3 null-mutant fish display reduced levels of skeletal-type EC coupling, suggesting that this adaptor protein is essential for stabilizing the Ca_V_1.1-RyR1 interaction [67,70] (reviewed recently by Flucher et al., [71]). In addition, this interaction is thought to be reinforced by junctophilins (JPs), which are a family of proteins (JP1–JP4) that act as columns for locking the SR to the plasma membrane and directly bind to Ca_V_1.1 [72,73,74,75]. Remarkably, in a recent study, Kurt Beam’s group was able to artificially reconstitute VGCR, in tsA201 cells expressing Ca_V_1.1, β_1a_, STAC3, RyR1, and JP2 [76].

### 4.3. Ca_V_1.1 Contributes to Keeping the RyR1s Closed

Most of the studies on skeletal EC coupling have focused on studying the RyR1 activation by Ca_V_1.1 (Figure 1A). In contrast, the possibility that Ca_V_1.1 also promotes RyR1 inhibition has been less investigated: the voltage sensor of Ca_V_1.1 could terminate the stimulus on RyR1, while returning to its deactivated state in response to membrane repolarization. A negative regulation has been indirectly observed, by mathematically deconvoluting the global Ca^2+^ transient (that is, applying equations based on a “removal model fit procedure” [77]). In particular, a square voltage pulse activates the permeability of SR to Ca^2+^, which reaches a maximum in a few milliseconds (peak) and then gradually decreases to a value that is slightly higher than the basal one (plateau). This decline is thought to reflect RyR1 inactivation by cytosolic Ca^2+^. However, when the membrane is repolarized, then the plateau quickly—and totally—ends, suggesting that voltage sensors returning to their deactivated state do, indeed, exert a negative regulation on RyR1 permeability [78]. 

The contribution of Ca_V_1.1 to keeping RyR1s closed, at resting membrane potentials, has also been demonstrated by assessing the production of sparks. For example, Zhou and coworkers demonstrated that while sparks occur spontaneously in the central regions of myotubes, they are practically absent in the periphery, where T-tubules and Ca_V_1.1 channels are present. In dysgenic myotubes, however, the spatial distribution of sparks is random, corroborating that the deactivated state of Ca_V_1.1 does, actually, inhibit the spontaneous opening of RyR1s [79]. Additional support for this view was reported recently [80]. 

### 4.4. Excitation-Coupled Calcium Entry (ECCE)

Given that the entry of Ca^2+^ is not required for skeletal-type EC coupling (Figure 1A, dashed pink line), one may ask if the Ca^2+^-conducting activity of Ca_V_1.1 has any biological significance, or remains only as an evolutionary remnant. In skeletal muscle fibers, the activation rate of Ca_V_1.1 is rather slow, and thus the corresponding I_Ca_ hardly influences the global myoplasmic [Ca^2+^]. The opposite may happen, however, under extreme experimental conditions. Particularly, it has been shown that long or repetitive sarcolemma depolarization produces an increase in myoplasmic [Ca^2+^], that depends on the entry of extracellular Ca^2+^ (termed excitation-coupled calcium entry or ECCE [81]). There is strong evidence suggesting that ECCE is due to the entry of Ca^2+^ via Ca_V_1.1, and this source of Ca^2+^ is thought to contribute to SR Ca^2+^ loading [82]. 

Interestingly, it has been reported that a point mutation in RyR1 (R163C) promotes an enhanced magnitude of ECCE, and this effect was interpreted to be of pathological relevance in malignant hyperthermia [83]. This view, however, requires more direct evidence, because ECCE is necessarily assessed under artificial conditions, e.g., blocking the SR Ca^2+^ release. Moreover, results from a recent study suggest that ECCE either: does not occur under physiological conditions or is irrelevant for muscle physiology. More precisely, the development and performance of skeletal muscles are unaltered in transgenic mice where the influx of Ca^2+^, via Ca_V_1.1, is eliminated [31]. 

## 5. Retrograde Signaling in Skeletal Muscle

Considering that Ca_V_1.1 physically controls the opening of RyR1 during skeletal-type EC coupling (orthograde coupling, Figure 1A), it should not be surprising that this physical interaction also modulates the function of Ca_V_1.1 (retrograde coupling, Figure 1A). In 1996, Kurt Beam’s group found evidence in favor of this retrograde signal. More precisely, they found that, in RyR1-null (dyspedic) myotubes, the I_Ca_ density is reduced to a nearly 10% of control values, in the face of practically no changes in the surface density of Ca_V_1.1 (inferred from measurements of intramembranous charge movement). Remarkably, this alteration was restored by transfecting dyspedic myotubes with cDNA encoding to RyR1, suggesting that the presence of RyR1 enhances the Ca^2+^ conducting activity of Ca_V_1.1 [29]. 

Moreover, results from whole-cell patch-clamp experiments indicate that RyR1s also regulate the following properties of Ca_V_1.1: activation kinetics, agonist DHP modulation, and divalent cation conductance [84]. At the single-channel level, the corresponding functional impact remains unexplored, but most likely involves an increase in P_o_ or channel conductance. Interestingly, it has also been shown that Ca^2+^ flowing through RyR1 enhances the steady-state expression of Ca_V_1.1 within days [85]. 

The precise segments or domains of Ca_V_1.1 that receive the retrograde signal of EC coupling have yet to be fully elucidated. Currently, it is generally accepted that the II–III loop is involved [54] [86], but controversy exists regarding which portions are the most critical. The interaction locus on RyR1 remains even more obscure, probably because of the colossal dimensions of this channel. Besides, the possible participation of accessory subunits contributes to generating more complexity. Indeed, the retrograde coupling is thought to depend on at least the following accessory proteins: β_1a_ and Stac3 [87,88]. 

Many lines of evidence suggest that certain inherited human myopathies originate from the expression of overactive or leaky RyR1s (reviewed recently by Fauré et al., [89] and Marty et al., [90]). Remarkably, Andronache and coworkers (2009) found that the altered gating of one of these RyR1 mutant proteins (Y522S) also exerts a retrograde influence on the function of Ca_V_1.1 [91]. They studied Ca^2+^ conductance and release (which reflect the activity of Ca_V_1.1 and RyR1), and reported that—in both cases—the steady-state voltage-dependence of inactivation was shifted towards more negative potentials (by nearly 10 mV, see also Vega et al., [92]); indicating that the mutation in RyR1 primes the voltage sensor of Ca_V_1.1 to inactivate. Moreover, because of this effect, the window of Ca^2+^ release is limited, which acts as a compensatory mechanism to counteract the leak of Ca^2+^ and SR depletion [91]. 

## 6. Cardiac EC Coupling

In contrast with Ca_V_1.1, which is almost exclusively expressed in skeletal muscle, Ca_V_1.2 predominates in cardiac myocytes. More specifically, ventricular myocytes primordially express Ca_V_1.2, but in atrial myocytes, the following channels have been detected (either at mRNA transcript or protein levels): Ca_V_3.1 and Ca_V_3.2 (low-voltage activated), Ca_V_1.2 and Ca_V_1.3 (L-type), and Ca_V_2.3 (R-type). An essential role for Ca_V_1.2 on cardiac EC coupling is amply recognized, while the relevance of the other channels remains elusive [1]. 

Pioneer experiments performed on skinned skeletal muscle fibers led to the conclusion that small increases in myoplasmic Ca^2+^ can trigger the release of Ca^2+^ from the SR (i.e., CICR [93,94]). Subsequently, CICR was also characterized in skinned cardiac myocytes, where its physiological role turned out to be superior to that of skeletal muscle. The influx of extracellular Ca^2+^ during an AP is insufficient to raise the global [Ca^2+^]_i_, to a level required to activate the contractile machinery, directly. Thus, CICR acts as an amplifying mechanism for bridging the gap between the entry of Ca^2+^ (via ion channels of the sarcolemma) and Ca^2+^-dependent activation of myofilaments [95]. 

Currently, it is widely accepted that an AP induces the influx of Ca^2+^ through Ca_V_1.2, and this Ca^2+^, in turn, directly activates RyR2 and thereby induces SR Ca^2+^ release (Figure 1B). The Ca_V_1.2 channels inactivate by both membrane depolarization and intracellular Ca^2+^, and these retrograde signals contribute to turning off the trigger of the system. Moreover, the ryanodine receptors also show inactivation, which, combined with partial SR depletion, inhibits the auto-regenerative CICR. The relaxation occurs when high levels of Ca^2+^ activate both the SERCA pump and the Na^+^-Ca^2+^ exchanger (NCX) and this, in turn, decreases the [Ca^2+^]_i_ to baseline levels (thus, deactivating the contractile machinery; for review, see Bers [24]). 

In both cardiac myocytes and skeletal muscle, the homeostasis of Ca^2+^ is remarkably well-regulated. For example, it has been reported that high levels of SR Ca^2+^ content correlate with an elevated frequency of spontaneous Ca^2+^ release [96] (recently referred to as “store-overload-induced Ca^2+^ release” (SOICR) [97]). This phenomenon could be explained by a higher Ca^2+^ conductance (inherent to the increased Ca^2+^ driving force), combined with the positive regulation of the P_o_ of RyRs by luminal SR Ca^2+^ [98,99,100]. Interestingly, the threshold for SOICR is reduced in many disease-linked mutations of both RyR1 and RyR2. Moreover, this functional defect contributes to explaining the overactive behavior of the corresponding mutant proteins, along with the human symptoms of central core disease (CCD), malignant hyperthermia (MH), and catecholaminergic polymorphic ventricular tachycardia (CPVT) [97,101]. The molecular bases underlying SOICR are not yet entirely clear. They most likely involve the binding of Ca^2+^ to the luminal sensing domains of the RyRs but could also imply binding of the ion to other molecules of the couplon. In addition, they could depend on another possible mechanism, termed “feed-through,” which consists of Ca^2+^ flowing via an open RyR and then binding to a cytosolic site on the same—or a neighboring—channel ([99], reviewed recently by Rios [102]). Regardless of the precise mechanism involved, it is clear that SOICR contributes to attenuating SR Ca^2+^ overload, and thus it may also prevent the development of premature beats and triggered arrhythmias [103]. Conversely, a partial SR depletion prevents the RyRs from being activated [104]. Thus, the SR is well-protected from being either overloaded or exhausted. Moreover, the Ca^2+^ that has entered to the cytosol can be both returned to the SR and extruded (via SERCA and NCX, respectively). Thus, in the steady state, the movements of Ca^2+^ are balanced and, thereby, the cell does not gain or lose Ca^2+^ (reviewed by Eisner et al., [105]). 

Nevertheless, the fine-tuning of EC coupling can be unbalanced by many conditions that undermine the function of the heart. In heart failure (HF), for example, the steady-state expression of SERCA and NCX is reduced and increased, respectively, while the P_o_ of RyR is enhanced. Accordingly, the SR is partially depleted, and the Ca^2+^ transients show both small amplitudes and low rate of decay [1]. 

In cardiac myocytes, the T-tubules are in continuity with longitudinal or axial elements (axial tubes or ATs), forming an interconnected, orderly network, termed a transverse-axial tubular system (TATS [106]). In small animals, this system is less prominent in atrial than in ventricular myocytes [107]. Accordingly, the Ca^2+^ transients of atrial myocytes are spatially inhomogeneous: they present a fast and a slow component, which are restricted to the cell periphery and center, respectively. The slow component is thought to be boosted by two mechanisms (in addition to a regular diffusion of CICR, known as “fire-diffuse-fire” [108]): (i) the presence of central couplons with highly phosphorylated RyR2 clusters, which show a more rapid release of Ca^2+^ (termed “super-hubs” [109]); (ii) it has been proposed that the RyR2s of centrally located couplons can be activated in tandem, i.e., via both cytosolic CICR and a parallel increase in the luminal SR Ca^2+^ content that reflects a higher SERCA activity, induced at the border of the cytosolic Ca^2+^ wave propagation. This model is known as the “fire-diffuse-uptake-fire” (FDUF) mechanism (reviewed by Blatter [110]). 

The cardiac myosin-binding protein-C (cMyBP-C) interacts with elements of the contractile machinery (i.e., actin and myosin filaments, in a phosphorylation-dependent manner) and is regulated by β-adrenergic stimulation. Interestingly, the phosphorylation of cMyBP-C enhances activation profiles nearby sites of Ca^2+^ binding to troponin C, and thereby accelerates the rate of cooperative cross-bridge recruitment (as reviewed by Moss et al [111]). Moreover, a recent study showed that cMyBP-C also physically interacts with RyR2s, and this interaction decreases the frequency of spontaneous Ca^2+^ oscillations, without significantly altering the rate of Ca^2+^ release or the amplitude/duration of Ca^2+^ transients [112]. These data were obtained in HEK293 cells, and thus their relevance for muscle pathophysiology remains to be elucidated. 

## 7. Retrograde Signaling in Cardiac Muscle

### Ca^2+^-Dependent Inactivation of Ca_V_1.2

The inactivation of Ca_V_1.2 by intracellular Ca^2+^ is a classic example of a retrograde signal at the T-tubule-SR junction (termed Ca^2+^-dependent inactivation or CDI, Figure 1B). CDI was first observed in *Paramecium*, and Ba^2+^ was incapable of reproducing this phenomenon [113]. Interestingly, prolonged depolarizations also inactivate VGCCs in a Ca^2+^-independent manner (known as voltage-dependent inactivation [114,115]). These two mechanisms prevent Ca^2+^ overload (which can be cytotoxic), and their contribution varies from one particular channel type to another (for review, see Cens et al., [116]). 

CDI occurs not only in response to a global increase in cytosolic [Ca^2+^] but also by the entry of calcium through a single channel (i.e., unitary current [117]). There is compelling evidence that calmodulin (CaM) represents the Ca^2+^ sensor for CDI [118,119,120]. CaM is a member of the EF-hand Ca^2+^ binding protein family and comprises two pairs of EF-hands, which are separated into N- and C-terminal lobes. The affinity for Ca^2+^ is lower in the former than in the latter (the corresponding K_d_ values are 1 µM and 0.1 µM, approximately [121]). Using CaM mutant proteins with disrupted Ca^2+^ binding in either N- (CaM_12_) or C-terminal (CaM_34_) lobes, Peterson and coworkers concluded that CDI is only supported by the CaM_12_ mutant. Thus, the C-terminal lobe of CaM was identified as the precise domain that binds Ca^2+^ for CDI [118]. 

On the other hand, it is currently accepted that the C-terminal tail of VGCCs is a critical element for attaching CaM and initiating the conformational change that results in CDI. This point has been amply investigated, and the corresponding evidence led Cens and collaborators to propose the following model [116]: The C-terminal tail contains three sites that, in the absence of Ca^2+^, interact and prevent CDI. When Ca^2+^ reaches CaM, this interaction is disrupted and, thereby, the brake for CDI is removed. The very essence of inactivation is then allowed to occur and involves the recruitment of the intracellular loop that links domains I–II (loop I–II). Conceivably, this loop may be acting on the pore by either an indirect constriction that may involve segments 6 (S6), or a direct obstruction [116]. Recently, it was shown that CDI could be eliminated by substituting a single amino acid of the pore region of human Ca_V_1.2 [122], corroborating the view that CDI culminates with changes in the pore configuration. 

In addition to CaM, other proteins of the triad (JPs and STACs, see Section 4.2) could also regulate both CDI and CICR. For example, the JPs are considered important for setting the distance that Ca^2+^ must travel from the sarcolemma to reach the Ca^2+^-binding sites of RyR2 [123]. Moreover, it has been demonstrated that the STACs drastically inhibit CDI, and this effect is likely due to an interaction between these accessory proteins and Ca_V_1.2 [68] (for review, see Flucher and Campiglio [71]). 

## 8. Auxiliary Ca^2+^ Signaling

### 8.1. SERCA

SERCA is an ~110-kDa transmembrane protein that belongs to the family of P-type ion-translocating ATPases and plays a pivotal role in the control of cytosolic Ca^2+^ concentration. In vertebrates, SERCA pumps are encoded by three different genes (SERCA1, 2, and 3). Each of them is transcribed in a tissue-specific manner, and alternate splicing results in at least ten isoforms. The SERCA2a isoform is expressed in both cardiac and slow-twitch skeletal muscles. Conversely, the fast-twitch skeletal muscle primordially expresses SERCA1a and SERCA1b (adult and fetal isoforms). The SERCA pump consists of a single polypeptide chain folded into four major domains: a transmembrane region (M) consisting of 10 helical segments (TM1 to TM10, which include two Ca^2+^-binding sites), and three cytosolic domains named A (actuator), N (nucleotide-binding), and P (phosphorylation). The active transport carried by SERCA can be described by a model termed E1-E2, which is based on a cycle that relies on a change in affinity for Ca^2+^-binding sites (from high–E1 to low–E2), and includes: phosphorylation by ATP, dephosphorylation, and reorientation of Ca^2+^ binding sites towards the SR lumen [124,125]. 

The SERCA activity can be regulated by single-pass transmembrane peptides such as phospholamban (PLB) and sarcolipin (SLN), which are differentially expressed—the former in ventricular myocytes and slow skeletal muscle, and the latter in fast-twitch skeletal muscle and atrial myocytes. The unphosphorylated forms of both PLB and SLN interact with and inhibit SERCA, whereas the phosphorylation relieves this inhibition. Indeed, the PKA-dependent phosphorylation of PLB is critical for the β-adrenergic stimulation of Ca^2+^ uptake and the ensuing increase in SR Ca^2+^ content [1]. Although the relevance of SLN is just beginning to be understood, this protein most likely also participates in β-adrenergic stimulation. Interestingly, PLB acts as an affinity inhibitor of SERCA for Ca^2+^, while SLN promotes the “uncoupling” of SERCA, which implies that the uptake of Ca^2+^ is reduced in the face of the unaltered hydrolysis of ATP. This uncoupling leads to increased heat production, and thus SLN may participate in thermogenesis and cold adaptation (reviewed by Bal et al., [126]). 

In the skeletal muscle of Duchenne muscular dystrophy (DMD) mouse models, the expression level of SLN is increased, and this alteration is thought to contribute to explaining a concomitant SERCA inhibition and Ca^2+^ overload [127]. Accordingly, reducing SLN expression in a mouse model of DMD (dystrophin/utrophin double mutant) results in enhanced SERCA function and mitigation of skeletal muscle and cardiac pathology (suggesting that reducing SLN levels is promising for treating DMD [128]). Besides, the downregulation of SLN leads to the restoration of a poor differentiation of dystrophic dog myoblasts [129]. 

Conversely, abating SLN in another mouse model (mdx) leads to adverse outcomes. In particular, low SLN levels inhibit the calcineurin signaling and thereby impair myogenesis and muscle regeneration [130]. Conceivably, this discrepancy could be explained by possible differences in the experimental models. Indeed, the cytosolic Ca^2+^ has the potential to promote both atrophy and hypertrophy, by stimulating protein breakdown and myogenesis (as described in Avila [131]). Thus, the outcome of stimulating SERCA may depend on the extent of the Ca^2+^ overload in each mouse model. Interestingly, in another inherited muscle disease (CCD), the upregulation of SERCA has also been proposed to counteract the altered Ca^2+^ homeostasis (albeit via PLB phosphorylation [92]). 

### 8.2. NCX

An increase in NCX activity, due to a rise in [Ca^2+^]_i_, can also be considered as a retrograde signal of the couplon (Figure 1B). There are three known mammalian NCX isoforms (NCX1, NCX2, and NCX3). Although NCX1 is also called the cardiac isoform, it is ubiquitously distributed. On the contrary, NCX2 is more abundant in the brain, and NCX3 is predominantly expressed in brain and skeletal muscle. Many splice variants have been detected, but only for NCX1 and NCX3. The NCX1 structure has been amply studied, and the corresponding conclusions in general apply to the other isoforms (by virtue of their high similitude in amino acid sequence, ~70%). The NCX1 was cloned by Nicoll et al. (1990), and consisted of 970 amino acids, from which a significant portion (550 amino acids) was termed the intracellular loop [132]. This loop divides the exchanger into two similar domains (N- and C-terminal), which are, in turn, composed of five transmembrane segments. Deleting the intracellular loop abolishes the allosteric modulation by Na^+^ and Ca^2+^, without losing the transporter activity. Each domain contains an α-repeat, which consists of regions with high intramolecular homology (oriented toward opposite sides of the sarcolemma) which participate in ion translocation [133,134,135]. 

In skeletal muscle, the role of the NCX is somewhat limited and probably restricted to pathological Na^+^ overload [136]. In marked contrast, the exchanger is of paramount relevance to cardiac muscle physiology: its role includes the regulation of both the intracellular levels of Na^+^ and Ca^2+^ and membrane potential. Specifically, its forward mode generates an inward current that contributes to extending the AP duration. On the other hand, the reverse mode is thought to be briefly triggered by the entry of Na^+^ during phase 0 of the AP, generating the early entry of Ca^2+^ that primes the subsequent activation of RyRs by I_Ca_. Under pathological conditions, however, a higher NCX activity contributes to generating electrical instability and arrhythmia (for it promotes the development of delayed afterdepolarizations and triggered activity) [1,137]. The extrusion of Ca^2+^ via NCX can be studied by analyzing the Ca^2+^ transient decay rate (in the absence of net RS Ca^2+^ uptake). This approach has led to the conclusion that, under physiological conditions, the NCX contributes to extruding Ca^2+^ by 7–28% (in rabbit and rat cardiac myocytes [138,139]). 

A number of heart conditions involve an altered function or expression of the exchanger, and the case of HF has been well-documented. For example, in a rabbit model of HF, both atrial and ventricular myocytes show a ~50–75% increase in NCX activity, which has been related to higher expression of NCX protein and mRNA [139,140]. Similar increases have also been observed in atrial myocytes derived from humans with atrial fibrillation and from corresponding animal models ([141] and references therein). 

### 8.3. Excitation–Metabolism Coupling

The mitochondria occupy nearly 10–30% of cell volume in cardiac myocytes and skeletal muscle fibers, and they are primarily distributed in regions nearby Ca^2+^ release units and contractile filaments. Their primary function is to produce ATP, and this activity is coupled with the SR Ca^2+^ release during EC coupling. In particular, the Ca^2+^-dependent activation of mitochondria matrix dehydrogenases stimulates the activity of the F_1_F_0_-ATPase. The entailed increase in ATP production helps to maintain an equilibrium with enhanced ATP expenditure (due to higher cross-bridge cycling and SERCA activity). The SR to mitochondrion signaling is thought to be bidirectional, because the mitochondria modulate the local redox environment of the couplon and thereby inhibit local SR Ca^2+^ release [142,143]. The ultimate consequences of this model remain elusive because reactive oxygen species (ROS) target a vast number of molecular targets, including molecular elements of EC coupling [144] and even the double modulation of a single protein (e.g., the stimulation and inactivation of RyRs [145]). Moreover, the NCX and SERCA are also considered ROS sensors, because redox modifications stimulate and inhibit their activity, respectively [146]. 

Experimental results show that electrical stimulation can elicit Ca^2+^ transients in the cytosolic and mitochondrial compartments (of both cardiac and skeletal muscle [147,148], reviewed by Franzini-Armstrong [149]). However, the magnitude of EC coupling-related mitochondrial Ca^2+^ transients differs between species or even during ontogeny (this could be explained by possible differences in the proximity of Ca^2+^ release units to mitochondrial Ca^2+^ transporters) [150]. In a reciprocal manner, the mitochondrial Ca^2+^ uptake attenuates the magnitude of the cytosolic Ca^2+^ transient [151,152]. 

Under physiological conditions, the mitochondrion to SR coupling involves a delicate balance of several processes. This balance, however, can be disrupted when the rate of ROS production overtakes antioxidant defenses, activating positive feedback mechanisms that ultimately lead to pathological conditions [153]. For example, in the skeletal muscle of mdx mice, a slightly elevated [Ca^2+^]_i_ induces higher ROS production, which in turn exacerbates Ca^2+^ overload [154]. Besides, redox modifications promote a leaky behavior of RyR1s, which may help in explaining a decline in force with aging [155]. Moreover, the ROS can also oxidize BH_4_—an essential cofactor of the nitric oxide synthase (NOS)—to BH_2_, and the resulting increase in BH_2_ to BH_4_ ratio promotes “NOS uncoupling,” which implies that the enzyme synthesizes superoxide anions, instead of nitric oxide (NO). Thus, the NOS uncoupling entails not only greater ROS production, but also the depletion of NO. The latter effect represents another level of complexity, because NO signaling also regulates the function of EC coupling-related proteins, via either direct S-nitrosylation or through the modulation of cGMP- and cAMP-dependent protein kinases (PKG and PKA) [144,156]. 

## 9. Concluding Remarks

The T-tubule-SR junction is a remarkable signaling hub whose specialized functions depend on a complex array of organelles, enzymes, and ion transport systems. The DHPRs play a preponderant role, orchestrating downstream and upstream signaling pathways for correct muscle function. Thus, changes in the concentration of Ca^2+^ are finely tuned by the activity of transporters and channels, whose expression is not only tissue-type specific but also properly targeted to distinct organelles of the junction. Many fundamental questions remain unsolved. For instance, what keeps the checkerboard pattern array of V and C RyRs in skeletal muscle? Is the Ca_V_1.1-RyR1 interaction necessary or perhaps even the sole requirement for explaining this particular array? Concerning the luminal concentration of SR Ca^2+^: which are the primary mechanisms for explaining the role of luminal Ca^2+^ on SR Ca^2+^ release? Moreover, which of these mechanisms are more influential under physiological and pathological conditions? Equally appealing could be the identification of novel factors that may participate in the excitation–metabolism coupling, as well as their precise impact on the function of EC-coupling-related proteins. Undoubtedly, studies solving these, and other similar questions will significantly advance the field of sarcolemma–SR symbiosis and, thereby, will also pave the route towards discovering novel therapeutic strategies for couplonopathies. 

## Abbreviations

AP	action potential
AT	axial tubes
[Ca^2+^]_i_	intracellular calcium concentration
CaM	calmodulin
CaMKII	calcium/calmodulin-dependent kinase II
CDI	Ca^2+^-dependent inactivation
CICR	Ca^2+^-induced Ca^2+^ release
PLB	phospholamban
cMyBP-C	cardiac myosin-binding protein-C
DHPR	dihydropyridine receptor
EC	excitation-contraction
ECCE	excitation-coupled calcium entry
ER	endoplasmic reticulum
FDUF	fire-diffuse-uptake-fire
HF	heart failure
I_Ca_	calcium current
TCA	tricarboxylic acid
JP	junctophilins
MCU	mitochondrial calcium uniporter
NCX	Na^+^-Ca^2+^ exchanger
NO	nitric oxide
NOS	nitric oxide synthase
MT	mitochondrion
PKA	cAMP-dependent protein kinase
PKG	cGMP-dependent protein kinase
Po	open probability
ROS	reactive oxygen species
RyR	ryanodine receptor
SERCA	sarcoplasmic reticulum Ca^2+^-ATPase
SLN	sarcolipin
SOICR	store-overload-induced Ca^2+^ release
SR	sarcoplasmic reticulum
TATS	transverse-axial tubular system
T-tubules	transverse tubules
VGCC	voltage-gated Ca^2+^ channel
VGCR	voltage-gated SR Ca^2+^ release
